# Risk Factors of Dilated Virchow-Robin Spaces Are Different in Various Brain Regions

**DOI:** 10.1371/journal.pone.0105505

**Published:** 2014-08-26

**Authors:** Changqing Zhang, Qidong Chen, Yilong Wang, Xingquan Zhao, Chunxue Wang, Liping Liu, Yuehua Pu, Xinying Zou, Wanliang Du, Yuesong Pan, Zixiao Li, Jing Jing, Dongxue Wang, Yang Luo, Ka Sing Wong, Yongjun Wang, on behalf of the Chinese IntraCranial AtheroSclerosis (CICAS) Study Group

**Affiliations:** 1 Department of Neurology, Beijing Tian Tan Hospital, Capital Medical University, Beijing, China; 2 Department of Nephrology, Beijing Tian Tan Hospital, Capital Medical University, Beijing, China; 3 Department of Medicine and Therapeutics, Prince of Wales Hospital, Chinese University of Hong Kong, Hong Kong, China; University Medical Center (UMC) Utrecht, Netherlands

## Abstract

**Background and Purpose:**

Few studies have reported on the risk factors of dilated Virchow-Robin Spaces (dVRS) in large samples of ischemic stroke patients. Little evidence exists regarding the relationship between dVRS and etiologic subtype of ischemic stroke or lacune. We aimed to investigate the risk factors associated with the severity of dVRS in a large sample of ischemic stroke patients.

**Methods:**

We consecutively enrolled 1,090 patients who experienced an ischemic stroke within the past seven days and underwent a 3.0 T MRI scan in the Chinese IntraCranial AtheroSclerosis Study (ICAS). Clinical data and cranial MRI information of patients included age, sex, vascular risk factors, dVRS, leukoaraiosis, lacune, and etiologic subtype of ischemic stroke. Analyses were performed regarding the risk factors associated with the severity of dVRS by univariate analysis and multivariable ordinal logistic regression analysis.

**Results:**

Through multivariable ordinal logistic regression analysis, age, the severity of leukoaraiosis, lacune, admission National Institutes of Health Stroke Scale (NIHSS) ≤3, and the severity of dVRS in the white matter (WM) and hippocampus (Hip) were correlated with the severity of dVRS in basal ganglia (BG); male, history of hypertension, admission NIHSS ≤3, and the severity of dVRS in BG and Hip were correlated with the severity of dVRS in WM; female, the severity of leukoaraiosis, admission NIHSS >3, small artery occlusion subtype of ischemic stroke, and the severity of dVRS in BG and WM were correlated with the severity of dVRS in Hip.

**Conclusion:**

dVRS is an indicator of cerebral small vessel diseases such as leukoaraiosis and lacune. However, the risk factors of dVRS differ in various brain regions.

## Introduction

Virchow-Robin spaces (VRS) are perivascular spaces that surround small arteries and arterioles [1]. When enlarged, these dilated Virchow-Robin spaces (dVRS) are commonly seen as punctate or linear hyperintensities on a T2-weighted brain magnetic resonance imaging (MRI). dVRS are widely detected in healthy individuals and in ischemic or hemorrhagic stroke patients [2]–[7]. Higher degree of dVRS were found to be independently associated with age, hypertension, and MRI marker of cerebral small vessel disease such as leukoaraiosis and lacune in stroke- and dementia-free community populations [2], whereas age, leukoaraiosis (LA), and deep cerebral microbleeds were associated with the severity of dVRS in patients with cerebral hemorrhage [7]. Additionally, it was found that age, lacunar stroke, and LA were related to the severity of dVRS in ischemic stroke patients [5]. Ischemic stroke patients had a higher prevalence of hypertension, diabetes, leukoaraiosis and lacune than community populations [8]–[10], however little information was available on the distribution and risk factors of dVRS in a large sample of ischemic stroke patients. There was also little evidence to support whether dVRS were significantly correlated with small vessel diseases such as leukoaraiosis and lacune, and with the severity or etiologic subtype of ischemic stroke. Therefore, the objective of our study was to analyze the distribution, severity, and risk factors of dVRS in a large sample of ischemic stroke patients.

## Methods and Materials

### Ethics Statement

This study was approved by the ethics committee of the Beijing Tian Tan Hospital of Capital Medical University and was performed in accordance with the guidelines of the Helsinki Declaration. After ethical approval of Tian Tan Hospital was obtained and approved by the other 21 participating hospitals (including Beijing Tongren Hospital of Capital Medical University; Shanghai Jiaotong University Affiliated Sixth People's Hospital; Shanghai Pudong New Area People's Hospital; Tianjin Huanhu Hosptial; Shanxi Provincial People's Hospital; The First Affiliated Hospital of Xiamen University; Xiangya Hospital Central-South University; Chengdu No. 3 People's Hospital; The First Affiliated Hospital of Jinan University; Guangzhou City Peoples First Hospital; Guangdong Hospital of Traditional Chinese Medicine; Handan Central Hospital; Handan First People's Hospital; Qingdao Municipal Hospital; The First Affiliated Hospital of Zhengzhou University; The First Affiliated Hospital of Zhejiang University; The First Affiliated Hospital of Wenzhou Medical College; Affiliated Kailuan Hospital, North China Coal Medical College; The First Affiliated Hospital of Beifang Medical College; Shijiazhuang Center Hospital; The first affiliated hospital of Hebei North University.), the ethical approval took effect automatically in each center. All patients or their legal representatives provide their written informed consent form.

### Subjects

ICAS was a prospective, multicenter, hospital-based study. Clinical and imaging data were collected from consecutive patients with ischemic stroke or transient ischemic attack (TIA) in 22 general hospitals in China. There were 2864 patients with noncardioembolic ischemic cerebrovascular diseases enrolled in ICAS. A total of 1090 subjects with ischemic stroke in seven hospitals underwent 3.0 T MR scanner and were enrolled in our study. The institutional review board of the participating hospitals approved the study. Each participant signed an informed consent statement.

Inclusion criteria included onset of symptoms within seven days, and age between 18 and 80 years old. Exclusion criteria were as follows: patients who were clinically unstable (i.e., they required close monitoring or were moribund), were disabled before admission (modified Rankin scale >2), and/or were unable to comply with MRI examination. We also excluded patients with cardioembolic risk factors (e.g., atrial fibrillation, valvular heart disease, postcardiac valve replacement, etc.) to rule out stroke patients due to cardioembolism. Full details of the design, and baseline information of the ICAS were previously published [11].

We evaluated clinical data (age, sex, and vascular risk factors) and the information of cranial MRI (dVRS, LA, lacune, and etiologic subtype of ischemic stroke) and analyzed the relationship between these factors and the severity of dVRS in different brain regions.

### Brain MRI

All 1090 patients in seven hospitals underwent imaging with the 3.0 T magnetic resonance (MR) scanner, including 3-dimensional time-of-flight MR angiography (3D TOF MRA, repetition time = 20–25 ms, echo time = 3.3–3.9 ms, flip angle = 15°–20°, slice thickness = 0.65–1.0 mm), and axial T2-weighted imaging (repetition time = 4500 ms; echo time = 84 ms), T1-weighted imaging (repetition time = 1200 ms; echo time = 11 ms), fluid-attenuated inversion recovery sequences (FLAIR, repetition time = 7000 ms; echo time = 94 ms), and diffusion-weighted imaging (DWI, repetition time = 3000 ms; echo time = 75 ms). All above sequences except MRA had 5 mm slice thickness and 1.5 mm interslice gap. MR images were transferred to a personal computer workstation and viewed using software (RadiAnt DICOM Viewer 1.0.4.4439, Medixant Ltd, Poznan, Poland). Rating of dVRS and LA were evaluated. Presence and quantities of lacune were also recorded.

Two radiologists who were blinded to patients' clinical information independently evaluated MRI data. Disagreements on the evaluations of dVRS, lacune, Fazekas score, and etiologic subtype of ischemic stroke were resolved through discussions.

### Rating of dVRS

**Figure 1:**
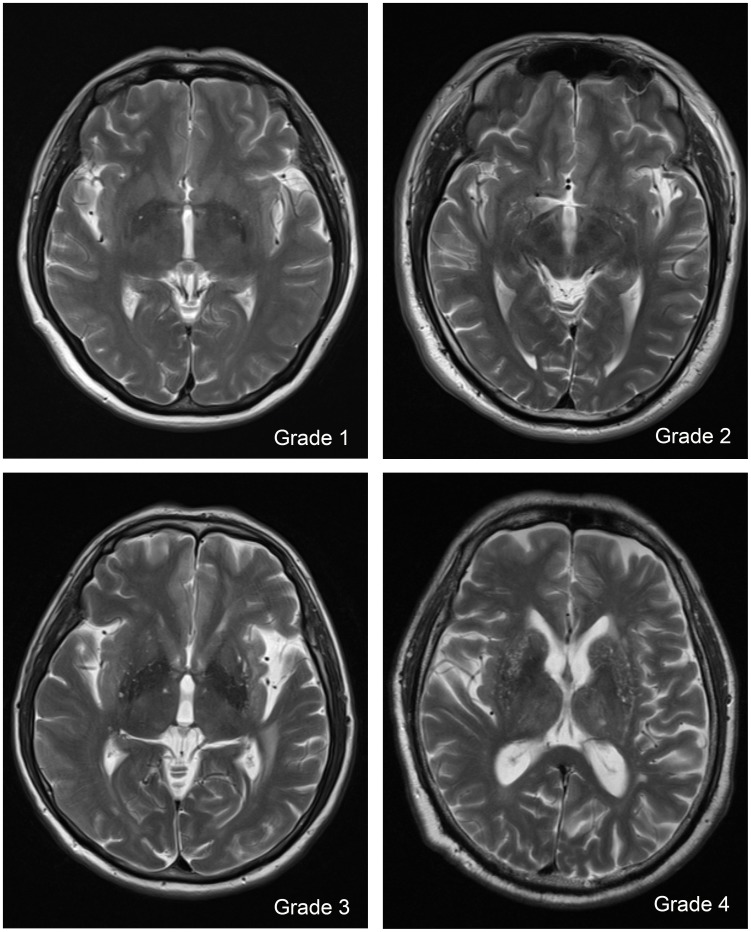
Severity score of dilated Virchow-Robin Spaces (dVRS) in basal ganglia.

The definition of dVRS was: 1) signal intensity of dVRS on T2-images being equivalent to that of the cerebrospinal fluid (CSF) (larger dVRS can be visible as hypointense lesions on FLAIR images, but generally without hyperintense rim); and 2) round, ovoid, or linear shape with a smooth margin, maximum diameter <3 mm and located in areas supplied by perforating arteries [2], [4]. For lesions fulfilling above criteria but maximum diameter ≥3 mm, only those with a typical vascular shape and following the orientation of perforating arteries were diagnosed as dVRS [2]. Severity of dVRS was rated in the following locations of brain:

In the basal ganglia (BG), dVRS were rated according to the number of dVRS in the slice containing the maximum amount of dVRS. The grades of dVRS were rated as follows: Grade 1: the number of dVRS <5; Grade 2: the number of dVRS, 5–10; Grade 3: the number of dVRS >10 but still countable; and Grade 4: the number of dVRS was infinite (Figure 1).

**Figure 2:**
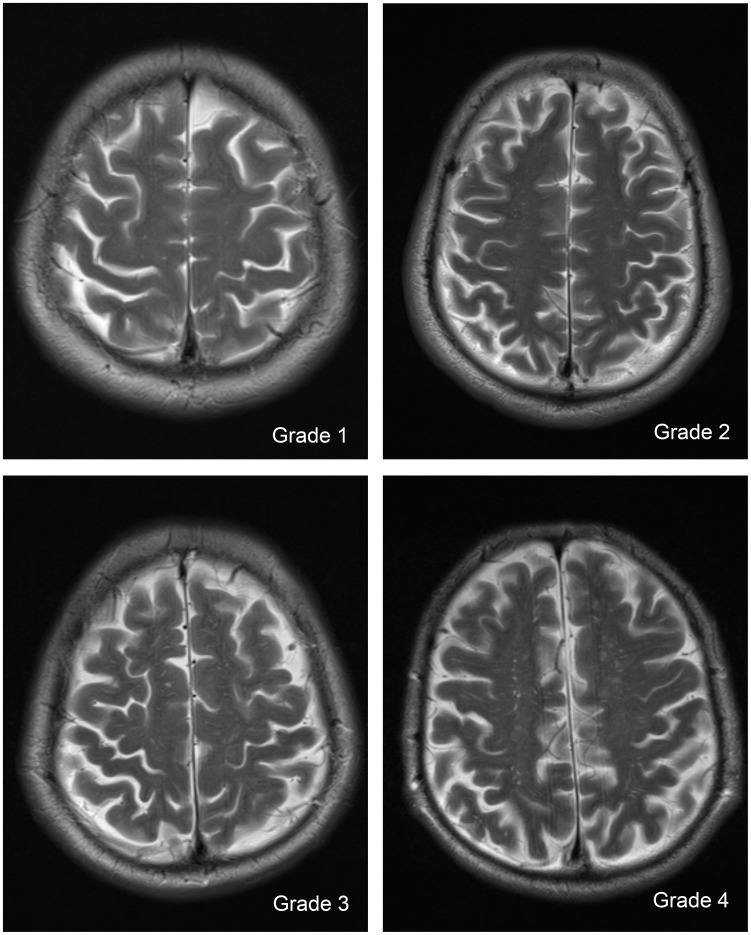
Severity score of dilated Virchow-Robin Spaces (dVRS) in white matter.

In the white matter (WM), dVRS were scored as follows: Grade 1 when the number of dVRS <10 in the entire WM slices; Grade 2 when the number of dVRS VRS in the entire WM slices but the number of dVRS <10 in any WM slice; Grade 3 when the number of dVRS ranged from 10 to 20 in the slice containing the maximum number of dVRS; and Grade 4 when the number of dVRS >20 in the slice containing the maximum number of dVRS (Figure 2) [2].

**Figure 3:**
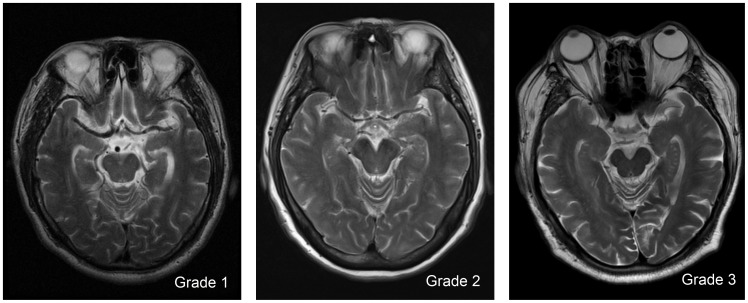
Severity score of dilated Virchow-Robin Spaces (dVRS) in hippocampus.

In the hippocampus (Hip), dVRS were scored according to number of dVRS in the total sections of hippocampus and parahippocampal gyrus. dVRS were rated as follows: Grade 1 when the number of dVRS <5; Grade 2 when the number of dVRS ranged from 5 to 10; and Grade 3, when the number of dVRS >10 (Figure 3).

### Other MRI Parameters

LA was defined as having a hyperintense lesion on FLAIR and T2 images, which was usually not seen on T1-weighted MRI or shown faint hypointensity at most. We rated LA with the Fazekas method [12]. Grades in periventricular white matter hyperintensities (PWMH) and deep white matter hyperintensities (DWMH) were evaluated separately and summed together as Fazekas score. Severity of LA was classified as Fazekas score <3 and Fazekas score ≥3.

Lacune were defined as lesions of 3 to 15 mm in size with the same signal characteristics as CSF on all sequences, with a hyperintense rim on the FLAIR sequence (when located supratentorially) [4], and were distinguished from dVRS by their typical wedged shape and surrounding hyperintensity on FLAIR [13].

Etiologic subtypes of ischemic stroke were classified according to the Stop Stroke Study Trial of Org 10172 in Acute Stroke Treatment (SSS-TOAST) classification criteria [14].

### Clinical Information Assessment

The clinical information collected from patients included age; sex; histories of hypertension, hyperlipidemia, ischemic stroke, stroke (including ischemic and hemorrhagic stroke), and coronary heart disease (defined as a history of myocardial infarction or angina pectoris); hypertension (defined as a history of hypertension or diagnosis at discharge); diabetes (defined as a history of diabetes or diagnosis at discharge); and NIHSS score at admission. Meanwhile, current or former smoking status (continuously smoking ≥1 cigarette a day for 6 months), and heavy drinking status (drinking >2 U/d on average for men or >1 U/d on average for women) were also recorded.

### Statistical Analyses

Continuous variables were summarized as mean value (standard deviation) or median (interquartile range) according to whether its distribution conformed to a normal distribution. Categorical variables were presented as percentages and absolute numbers. Analysis of variance was used for comparison of continuous variables with normal distribution, and Kruskal-Wallis test was used for comparison of continuous variables with non-normal distribution. Chi-squared (χ^2^) test was used for comparison of categorical variables. The baseline potential risk factors and their crude distribution were presented according to the severity of dVRS. Multivariable ordinal logistic regression analysis was used to identify predictors of increasing dVRS severity in BG, WM, and Hip. All parameters that were significant in the univariate analysis at *P*<0.05 level or likely to have pathophysiological influence on dVRS were included in the multivariable ordinal logisitic regression model. Interobserver agreement of the dVRS grading was assessed by using kappa (K) test to quantify the level of agreement. All probability values were 2-tailed; *P*<0.05 was considered to be statistically significant. All analyses were performed using SAS Version 9.1 (SAS Institute, Inc, Cary, NC).

## Results

Baseline characteristics of the 1090 patients are shown in Table 1. There were 783 men (71.8%) and 307 women (28.2%). As for etiologic subtype of ischemic stroke, 369 patients (33.9%) were classified as being in the small-artery occlusion (SAO) group, whereas 721 (66.1%) were in the large artery atherosclerosis (LAA) group. Among the 1090 patients, 546 underwent the evaluation of size of dVRS, 436 of them (79.9%) had dVRS of more than or equal to 3 mm in maximum diameter.

**Table 1:** Crude Distribution of Potential Risk Factors Across the Grades of dVRS in BG*.

	Total (N = 1090)	Grade 1 (N = 200)	Grade 2 (N = 575)	Grade 3 (N = 247)	Grade 4 (N = 68)	*P* value
Mean age, years (SD)	60.4(11.2)	56.0(11.3)	59.2(10.5)	64.2(10.8)	70.5(6.5)	<0.0001
Age ≥65 years	38.7(422)	23.5(47)	32.3(186)	52.2(129)	88.2(60)	<0.0001
Gender (Male)	71.8(783)	73.0(146)	71.3(410)	72.1(178)	72.1(49)	0.97
Current or previous smoker	55.1(601)	62.0(124)	57.7(332)	48.6(120)	36.8(25)	<0.0001
heavy drinker	6.3(69)	5.0(10)	6.8(39)	6.9(17)	4.4(3)	0.72
Hypertension	79.1(861)	74.0(148)	79.6(457)	79.8(197)	86.8(59)	0.13
History of hypertension	66.9(722)	59.6(118)	66.4(378)	70.6(173)	79.1(53)	0.01
Diabetes mellitus	37.6(409)	41.5(83)	37.5(215)	36.7(90)	30.9(21)	0.44
Hyperlipidemia	17.0(164)	15.3(27)	20.0(103)	13.9(29)	8.3(5)	0.04
Ischemic heart diseases	9.8(102)	10.2(20)	10.1(56)	9.7(22)	6.3(4)	0.82
previous ischemic stroke	26.3(286)	19.5(39)	25.0(144)	32.1(79)	35.3(24)	0.006
previous stroke	27.7(302)	22.0(44)	26.1(150)	33.3(82)	38.2(26)	0.009
NIHSS score at admission ≤3	42.2(457)	33.0(66)	42.3(243)	46.7(113)	52.2(35)	0.008
Leukoaraiosis	95.9(1045)	91.0(182)	95.5(549)	99.6(246)	100(68)	<0.0001
Fazekas score ≥3	50.2(547)	31.5(63)	43.8(252)	68.4(169)	92.6(63)	<0.0001
Lacune	44.7(468)	34.7(67)	41.2(228)	53.8(126)	71.2(47)	<0.0001
Number of Lacune, median (IQR)	0(0–2)	0(0–1)	0(0–1)	1(0–3)	2(0–4)	<0.0001
Small-artery occlusion subtype†	33.9(369)	27.0(54)	35.0(201)	35.6(88)	38.2(26)	0.14

Abbreviations: dVRS, dilated Virchow-Robin Spaces; BG, basal ganglia; SD, standard deviation; NIHSS, National Institutes of Health stroke scale; IQR, interquartile range.

*All data are presented as percentage (no.) unless otherwise indicated.

† It is contrary to large-artery atherosclerosis subtype of ischemic stroke according to Stop Stroke Study Trial of Org 10172 in Acute Stroke Treatment (SSS-TOAST) classification criteria.

All of 1090 patients had dVRS, the percentages and numbers of 0–4 grade dVRS in BG, WM and Hip are demonstrated in Table 1, Table 2, Table 3 and Table 4. Mean dVRS scores in BG, WM, and Hip were 2.17, 2.55, and 1.16 respectively. Pearson correlation coefficient was 0.23 (*P*<0.0001) between BG and WM dVRS, 0.24 (*P*<0.0001) between BG and Hip dVRS, and 0.28 (*P*<0.0001) between WM and Hip dVRS. The proportion (number) of Grade 0–3 of PWMH was 5.5% (60), 46.1% (502), 35.2% (384) and 13.2% (144) respectively, the proportion (number) of Grade 0–3 of DWMH was 15.4% (168), 48.5% (529), 28.0% (305) and 8.1% (88) respectively. Mean Fazekas score was 2.85. Among the 1090 patients, 44 patients' images of lacune could not be evaluated due to the poor quality of MR images. The mean number of lacune was 1.5. Most patients 1069 (98.1%) had symmetric dVRS in bilateral hemisphere, only 21 (1.9%) patients had noticeable inter-hemispheric difference in the severity of dVRS. All these 21 patients had significant stenosis or occlusion of ipsilateral internal carotid artery or middle cerebral artery.

**Table 2:** Crude Distribution of Potential Risk Factors Across the Grades of dVRS in WM*.

	Total (N = 1090)	Grade 0 (N = 9)	Grade 1 (N = 181)	Grade 2 (N = 334)	Grade 3 (N = 334)	Grade 4 (N = 232)	*P* value
Mean age, years (SD)	60.4(11.2)	70.6(10.7)	61.1(12.4)	59.9(11.5)	60.5(10.6)	60.2(10.4)	0.07
Age ≥65 years	38.7(422)	77.8(7)	44.2(80)	37.1(124)	38.0(127)	36.2(84)	0.06
Gender (Male)	71.8(783)	77.8(7)	67.4(122)	69.2(231)	73.7(246)	76.3(177)	0.21
Current or previous smoker	55.1(601)	44.4(4)	59.7(108)	53.9(180)	55.1(184)	53.9(125)	0.69
Heavy drinker	6.3(69)	0(0)	5.5(10)	4.8(16)	6.6(22)	9.1(21)	0.28
Hypertension	79.1(861)	88.9(8)	71.8(130)	78.7(262)	78.1(261)	86.2(200)	0.009
History of hypertension	66.9(722)	77.8(7)	53.9(97)	65.0(212)	67.9(226)	77.9(180)	<0.0001
Diabetes mellitus	37.6(409)	22.2(2)	37.6(68)	34.7(116)	39.6(132)	39.6(91)	0.55
Hyperlipidemia	17.0(164)	22.2(2)	19.4(31)	13.6(40)	17.5(51)	19.4(40)	0.38
Ischemic heart diseases	9.8(102)	22.2(2)	10.2(18)	5.9(19)	13.5(43)	9.3(20)	0.02
Previous ischemic stroke	26.3(286)	55.6(5)	24.9(45)	24.0(80)	28.5(95)	26.3(61)	0.20
Previous stroke	27.7(302)	66.7(6)	26.5(48)	25.4(85)	30.0(100)	27.2(63)	0.07
NIHSS score at admission ≤3	42.2(457)	33.3(3)	30.0(54)	39.5(132)	44.9(149)	52.2(119)	<0.0001
Leukoaraiosis	95.9(1045)	100(9)	92.8(168)	95.8(320)	96.7(323)	97.0(225)	0.20
Fazekas score ≥3	50.2(547)	100(9)	49.7(90)	46.4(155)	50.0(167)	54.3(126)	0.01
Lacune	44.7(468)	62.5(5)	47.4(82)	43.3(138)	43.6(140)	45.8(103)	0.73
Number of lacune, median (IQR)	0(0–2)	1.5(0–6.3)	0(0–2)	0(0–2)	0(0–2)	0(0–2)	0.58
Small-artery occlusion subtype†	33.9(369)	33.3(3)	36.5(66)	37.1(124)	27.8(93)	35.8(83)	0.10

Abbreviations: dVRS, dilated Virchow-Robin Spaces; WM, white matter; SD, standard deviation; NIHSS, National Institutes of Health stroke scale; IQR, interquartile range.

*All data are presented as percentage (no.) unless otherwise indicated.

† It is contrary to large-artery atherosclerosis subtype of ischemic stroke according to Stop Stroke Study Trial of Org 10172 in Acute Stroke Treatment (SSS-TOAST) classification criteria.

**Table 3:** Crude Distribution of Potential Risk Factors Across the Grades of dVRS in WM after removing 149 patients with Grade 3 PWMH or Grade 3 DWMH*.

	Total (N = 941)	Grade 1 (N = 139)	Grade 2 (N = 293)	Grade 3 (N = 299)	Grade 4 (N = 210)	*P* value
Mean age, years (SD)	59.2(11.0)	58.2(12.2)	58.8(11.4)	59.6(10.5)	59.7(10.5)	0.47
Age ≥65 years	32.8(309)	32.4(45)	31.7(93)	33.4(100)	33.8(71)	0.96
Gender (Male)	72.2(679)	71.9(100)	67.6(198)	73.6(220)	76.7(161)	0.14
Current or previous smoker	56.5(532)	67.6(94)	53.2(156)	56.2(168)	54.3(114)	0.03
Heavy drinker	7.0(66)	7.2(10)	4.8(14)	7.4(22)	9.5(20)	0.23
Hypertension	78.8(741)	67.6(94)	78.8(230)	78.6(235)	86.7(182)	<0.0001
History of hypertension	66.0(614)	44.9(62)	65.0(186)	68.1(203)	78.0(163)	<0.0001
Diabetes mellitus	39.1(367)	38.8(54)	36.9(108)	40.5(121)	40.4(84)	0.80
Hyperlipidemia	17.2(143)	20.0(25)	14.0(36)	17.2(45)	19.9(37)	0.32
Ischemic heart diseases	9.6(87)	8.8(12)	6.7(19)	12.8(37)	9.7(19)	0.10
Previous ischemic stroke	23.2(218)	18.0(25)	21.2(62)	27.5(82)	23.3(49)	0.12
Previous stroke	24.4(229)	19.4(27)	22.2(65)	29.2(87)	23.8(50)	0.09
NIHSS score at admission ≤3	42.4(397)	26.1(36)	40.6(119)	45.3(135)	51.7(107)	<0.0001
Leukoaraiosis	95.2(896)	90.6(126)	95.2(279)	96.3(288)	96.7(203)	0.04
Fazekas score ≥3	42.3(398)	34.5(48)	38.9(114)	44.1(132)	49.5(104)	0.02
Lacune	40.3(366)	35.1(47)	39.6(112)	41.5(120)	42.9(87)	0.51
Number of lacune, median (IQR)	0(0–1)	0(0–1)	0(0–2)	0(0–1)	0(0–2)	0.34
Small-artery occlusion subtype†	31.9(300)	33.8(47)	34.8(102)	26.8(80)	33.8(71)	0.15

Abbreviations: dVRS, dilated Virchow-Robin Spaces; WM, white matter; SD, standard deviation; NIHSS, National Institutes of Health stroke scale; PWMH, periventricular white matter hyperintensities; DWMH, deep white matter hyperintensities. IQR, interquartile range.

*All data are presented as percentage (no.) unless otherwise indicated.

† It is contrary to large-artery atherosclerosis subtype of ischemic stroke according to Stop Stroke Study Trial of Org 10172 in Acute Stroke Treatment (SSS-TOAST) classification criteria.

**Table 4:** Crude Distribution of Potential Risk Factors Across the Grades of dVRS in Hip*.

	Total (N = 1090)	Grade 0 (N = 174)	Grade 1 (N = 616)	Grade 2 (N = 254)	Grade 3 (N = 46)	*P* value
Mean age, years (SD)	60.4(11.2)	58.9(11.4)	60.4(11.4)	61.0(10.8)	63.1(9.2)	0.09
Age ≥65 years	38.7(422)	28.7(50)	39.4(243)	42.5(108)	45.7(21)	0.02
Gender (Male)	71.8(783)	75.3(131)	73.2(451)	67.7(172)	63.0(29)	0.14
Current or previous smoker	55.1(601)	62.1(108)	55.8(344)	50.4(128)	45.7(21)	0.06
Heavy drinker	6.3(69)	8.6(15)	6.3(39)	5.1(13)	4.3(2)	0.48
Hypertension	79.1(861)	74.1(129)	78.0(480)	83.5(212)	87.0(40)	0.05
History of hypertension	66.9(722)	57.2(99)	66.7(406)	72.1(181)	78.3(36)	0.004
Diabetes mellitus	37.6(409)	41.4(72)	35.1(216)	41.5(105)	35.6(16)	0.22
Hyperlipidemia	17.0(164)	17.9(27)	17.3(95)	17.1(38)	10.0(4)	0.68
Ischemic heart diseases	9.8(102)	8.2(14)	11.1(65)	7.5(18)	11.6(5)	0.36
Previous ischemic stroke	26.3(286)	23.7(41)	25.8(159)	27.2(69)	37.0(17)	0.32
Previous stroke	27.7(302)	25.4(44)	26.8(165)	29.5(75)	39.1(18)	0.25
NIHSS score at admission ≤3	42.2(457)	40.2(70)	43.8(268)	41.0(103)	34.8(16)	0.55
Leukoaraiosis	95.9(1045)	93.1(162)	95.3(587)	98.4(250)	100(46)	0.02
Fazekas score ≥3	50.2(547)	37.4(65)	47.6(293)	61.8(157)	69.6(32)	<0.0001
Lacune	44.7(468)	40.4(67)	42.3(250)	52.8(130)	48.8(21)	0.02
Number of lacune, median (IQR)	0(0–2)	0(0–2)	0(0–2)	1(0–2)	0(0–4)	0.01
Small-artery occlusion subtype†	33.9(369)	26.4(46)	33.8(208)	37.8(96)	41.3(19)	0.07

Abbreviations: dVRS, dilated Virchow-Robin Spaces; Hip, hippocampus; SD, standard deviation; NIHSS, National Institutes of Health stroke scale; IQR, interquartile range. *All data are presented as percentage (no.) unless otherwise indicated.

† It is contrary to large-artery atherosclerosis subtype of ischemic stroke according to Stop Stroke Study Trial of Org 10172 in Acute Stroke Treatment (SSS-TOAST) classification criteria.

Interrater agreement from the two assessors for the ratings of dVRS in BG, WM, and Hip were high (*K* value = 0.68, 0.67, and 0.79 respectively). Interrater agreement from the two assessors for the ratings of PWMH and DWMH were also high (*K* value = 0.88, 0.86 respectively).

### Associations of dVRS in BG

In univariate analysis, the severity of dVRS in BG was associated with increasing age, never smoking, history of hypertension, history of ischemic stroke, history of stroke, the presence of LA, Fazekas score ≥3, the presence of lacune, the number of lacune, and admission NIHSS score ≤3 (Table 1). In multivariable ordinal logistic regression analysis, only age ≥65 years (*P*<0·0001), Fazekas score ≥3 (*P*<0·0001), the presence of lacune (*P* = 0·02), admission NIHSS score ≤3 (*P* = 0·02), and the severity of dVRS in WM and Hip (*P*<0·0001) remained statistically significant in association with the severity of dVRS in BG (Table 5).

**Table 5:** Multivariable Ordinal Logistic Regression Analysis for Risk Factors Associated with Severity of dVRS in BG, WM*, and Hip.

	dVRS in BG (N = 1028)		dVRS in WM* (N = 894)		dVRS in Hip (N = 1028)	
	*P* value	OR(95% CI)	*P* value	OR(95% CI)	*P* value	OR(95% CI)
Age ≥65 years	<0.0001	2.79(2.13–3.67)	0.14	0.81(0.62–1.07)	0.08	1.08(0.82–1.43)
Gender (Male)	0.23	1.18(0.90–1.55)	0.03	1.36(1.03–1.80)	0.01	0.71(0.54–0.93)
History of hypertension	0.39	1.13(0.86–1.47)	<0.0001	2.01(1.54–2.63)	0.30	1.15(0.88–1.51)
NIHSS score at admission ≤3	0.02	1.35(1.06–1.73)	<0.0001	1.86(1.45–2.39)	0.004	0.69(0.54–0.89)
Fazekas score ≥3	<0.0001	2.16(1.63–2.87)	0.93	0.99(0.75–1.30)	0.003	1.55(1.17–2.05)
Lacune	0.02	1.36(1.05–1.78)	0.24	0.85(0.65–1.11)	0.65	1.06(0.81–1.39)
Small-artery occlusion subtype†	0.17	1.20(0.93–1.54)	0.08	0.79(0.61–1.02)	0.006	1.44(1.11–1.86)
dVRS score in WM	<0.0001	1.51(1.33–1.72)			<0.0001	1.73(1.52–1.96)
dVRS score in Hip	<0.0001	1.46(1.23–1.74)	<0.0001	1.92(1.61–2.30)		
dVRS score in BG			<0.0001	1.80(1.51–2.16)	<0.0001	1.43(1.21–1.70)

Abbreviations: dVRS, dilated Virchow-Robin Spaces; BG, basal ganglia; WM, white matter; Hip, hippocampus; NIHSS, National Institutes of Health stroke scale; PWMH, periventricular white matter hyperintensities; DWMH, deep white matter hyperintensities.

*The 134 patients with grade 3 PWMH or grade 3 DWMH were excluded.

† It is contrary to large-artery atherosclerosis subtype of ischemic stroke according to Stop Stroke Study Trial of Org 10172 in Acute Stroke Treatment (SSS-TOAST) classification criteria.

### Associations of dVRS in WM

In order to rule out the influence of white matter hyperintensities (WMH) with regard to the evaluation of dVRS, we carried out univariate and multivariable analyses after removing patients with grade 3 PWMH or grade 3 DWMH, and found that history of hypertension, admission NIHSS score ≤3, the presence of LA, and Fazekas score ≥3 significantly related with more serious dVRS in WM in univariate analyses (Table 3). In multivariable ordinal logistic regression analysis, male (*P* = 0·03), history of hypertension (*P*<0·0001), admission NIHSS score ≤3 (*P*<0·0001), and the severity of dVRS in BG and Hip (*P*<0·0001) remained significant in association with the severity of dVRS in WM (Table 5).

### Associations of dVRS in Hip

In univariate analysis, the severity of dVRS in Hip was associated with increasing age, never smoking, history of hypertension, the presence of LA, Fazekas score ≥3, and the presence of lacune (Table 4). In multivariable ordinal logistic regression analysis, female (*P* = 0.01), admission NIHSS score >3 (*P* = 0·004), Fazekas score ≥3 (*P* = 0·003), SAO subtype stroke (*P* = 0.006), and the severity of dVRS in BG and WM (*P*<0·0001) remained significant in association with the severity of dVRS in Hip (Table 5).

## Discussion

This study, performed in 1090 ischemic stroke patients, showed that dVRS were frequent (100%) in noncardioembolic ischemic stroke patients. dVRS most commonly occured in BG, and then in WM and Hip.

However, multivariable ordinal logistic regression analysis showed that the risk factors of dVRS varied according to the regions. Increasing age was only significantly associated with dVRS in BG; hypertension was an independent risk factor for dVRS in WM but not in BG and Hip; males had a higher risk for more severe dVRS in WM, whereas females had a higher risk for more severe dVRS in Hip relatively; severe leukoaraiosis (Fazekas score ≥3) was significantly associated with increasing severity of dVRS in BG and Hip, but not with that in WM; the presence of lacune was positively correlated with more severe dVRS in BG, but not with that in WM or Hip; SAO subtype stroke was only significantly related with more severe dVRS in Hip.

Risk factors for dVRS in various brain regions were different in our study, several reasons may account for this. First, regional variations in severity have been observed for fibrohyaline thickening, lipohyalinosis, and cerebral amyloid angiopathy (CAA) within the microvasculature during ageing [15], which may result in the differences of the severity of vessel wall permeability and dVRS in various regions of brain [2], [16]. Therefore, the underlying microvascular pathological changes of dVRS in different brain regions may be different. Increasing age is an important risk factor for CAA [17], whereas hypertension is the main cause of fibrohyaline thickening and lipohyalinosis [18]. These factors may be one possible reason for the difference in risk factors of dVRS in various brain regions. Second, the exact mechanisms of dVRS are still unknown. Segmental necrotizing angiitis of the arteries which caused increased permeability of the arterial wall, gradual leakage of the interstitial fluid from the intracellular compartment to the pial space, fibrosis and obstruction of Virchow-Robin Spaces along the small arteries, and disturbance of the drainage route of interstitial fluid are some hypotheses that have been presented [1]. dVRS in specific brain regions may be caused by one mechanism or concurrence of several mechanisms mentioned above, this may be another possible reason for the difference in risk factors of dVRS in various brain regions. However, because the pathological studies about dVRS are very limited, all these hypotheses need further histopathological studies to verify them.

Rouhl et al [4] found that the severity of dVRS in BG was positively correlated with the severity of LA. We also found that severe LA was an independent risk factor for dVRS in BG and Hip. Blood-brain barrier dysfunction is a possible mechanism of LA [16]. Meanwhile, gradual leaking of the interstitial fluid to the pial space due to blood-brain barrier dysfunction is also a possible mechanism of dVRS [19]. This may be the reason why LA and dVRS often co-exist in pathology studies [20] and are significantly relevant in MRIs.

The association between dVRS and lacune appeared to be stronger in BG than in WM [2], [4]. We also found that the presence of lacune was positively correlated with more severe dVRS in BG. Arteriolosclerosis is an important characteristic of lacune in pathology [21], and dVRS is also related to the presence of arteriolosclerosis [22]. Meanwhile, lacunar stroke and dVRS were also associated with diffuse blood-brain barrier dysfunction [23]. Hence, arteriolosclerosis and blood-brain barrier dysfunction may be the common mechanisms of lacune and dVRS, and lead to the significant correlation between them.

Our study specifically investigated the association between dVRS and SSS-TOAST etiologic subtype of ischemic stroke, and found that SAO subtype stroke was significantly correlated with more severe dVRS in Hip. The result further supports that dVRS is another indicator of small vessel diseases (SVD). The associations between the severity of dVRS and the presence of old lacunes, severity of LA and SAO subtype stroke suggested that the development of dVRS could be the consequence of an underlying SVD. These findings are in agreement with previous pathological and MRI results [3], [22], [24]. Increased blood-brain permeability has been observed in patients with LA [25], lacune and dVRS [23], and may be an important common mechanism of cerebral SVD. This may explain why LA, lacune, and dVRS are significantly correlated with with each other.

The strengths of our study included the use of carefully standardized research methods and a large sample size. Our study had several limitations. First, lacune could not be evaluated in 44 patients due to the poor quality of MR images. Second, because some patients' clinical information was incomplete, only 1028 of 1090 patients underwent multivariable logistic regression analysis. Third, this was a cross section study, further prospective studies were needed to confirm the cause and effect relationship between these risk factor and dVRS. Lastly, because the pathological studies about dVRS were very few and limited, further histopathological studies are needed to account for the regional variations in risk factors of dVRS.

## Conclusions

Overall, our study demonstrated that the severity of dVRS in certain brain regions was significantly correlated with age, history of hypertension, the severity of LA, the presence of lacune, and SAO subtype stroke; and dVRS was another MRI marker of SVD. However, there were some regional variations among the risk factors associated with the severity of dVRS, which indicates that the developmental mechanisms of dVRS may differ in various brain regions.
